# Effects of multi-disciplinary team nursing model combined with high-fiber dietary intervention on blood glucose level and nutritional status in elderly type 2 diabetes patients: a prospective clinical trial

**DOI:** 10.3389/fmed.2025.1631466

**Published:** 2025-10-08

**Authors:** Haiyun Wang, Mengping Yan, Hongyin Wang, Lingbo He, Yingzi Zhang

**Affiliations:** ^1^Department of General Medicine, Beijing Friendship Hospital, Capital Medical University, Beijing, China; ^2^Department of Emergency, Beijing Friendship Hospital, Capital Medical University, Beijing, China; ^3^Department of Emergency, Wangjing Hospital, China Academy of Chinese Medical Sciences, Beijing, China

**Keywords:** diabetes, multi-disciplinary team nursing, high-fiber dietary intervention, blood glucose, nutritional status

## Abstract

**Objective:**

This study was a prospective clinical trial, aimed to explore the effects of multi-disciplinary team (MDT) nursing model combined with high-fiber dietary intervention on blood glucose level and nutritional status in elderly diabetes patients.

**Methods:**

One hundred and sixty-eight elderly type 2 diabetes patients admitted from April 2020 to April 2023 were allocated into intervention group and control group. Patients in the control group accepted routine nursing + basic nutritional therapy. On the basis of the control group, patients in the intervention group adopted MDT nursing + high-fiber dietary intervention. Both groups received an 8-week intervention. The blood glucose indexes, incidence of complications, quality of life, nutritional status, negative emotions, sleep quality along with patient satisfaction were compared in two groups.

**Results:**

After 8 weeks of intervention, when comparing with the control group, the fasting blood glucose, 2 h postprandial blood glucose as well as glycosylated hemoglobin in the intervention group were lower (*p* < 0.05), the incidence of complications in the intervention group was lower (*p* < 0.05), the scores of body function, psychological state, social function along with role function in the intervention group were higher (*p* < 0.01), the ALB and TP levels in the intervention group were higher (*p* < 0.05), the SAS and SAS scores in the intervention group were lower (*p* < 0.05), the PSQI score in the intervention group were lower (*p* < 0.05), and the patient satisfaction in the intervention group was higher (*p* < 0.05).

**Conclusion:**

MDT model in combination with high-fiber dietary intervention can control blood glucose level, promote nutritional status, decrease the incidence of complications along with promote the quality of life in elderly diabetes patients.

## 1 Introduction

Diabetes mellitus belongs to a group of endocrine and metabolic diseases featured by chronic persistent hyperglycemia caused by insulin deficiency or abnormal insulin biological function (1). Its typical clinical manifestations are polydipsia, polyphagia, polyuria, and emaciation (2). At present, with the continuous progress of social economy and the improvement of life quality, the incidence of diabetes is increasing, and the disease and its complications seriously endanger the life safety of patients (3).

According to the WHO classification, diabetes can be separated into type 1 diabetes and type 2 diabetes (4). Type 2 diabetes is also known as adult-onset diabetes, the onset age is generally 35–40 years old, and it accounts for more than 90% of diabetes patients, which are mainly elderly patients (5). Clinical studies believe that the treatment of diabetes should be mainly to control the development of the disease and improve the blood sugar level of the body, and during the conventional treatment, it is also necessary to closely monitor the changes of diabetes in patients to reduce the risk of later complications (6). However, the treatment period of diabetes is long and difficult to cure, so it is necessary to assist the health care work in the treatment process to improve the treatment effect as well as improve the prognosis of patients (7).

The traditional nursing mode is mainly specialized nursing of diabetes mellitus, including publicity and education of diabetes disease knowledge, monitoring of patients’ blood sugar and medication guidance (8). However, elderly patients are often accompanied by other diseases, as well as decreased resistance, unbalanced nutrition and other reasons, leading to difficult nursing and easy to produce psychological problems (9). Therefore, single-department nursing is not effective in elderly patients with diabetes.

Multi-disciplinary team (MDT) nursing is a nursing mode that takes multi-center clinical disciplines as the starting point and multi-disciplinary diabetes nursing team as the core, and supplements the shortcomings of traditional specialized nursing with the help of team joint management mode, so as to formulate targeted nursing plans for patients and meet their life needs (10). On the basis of multi-disciplines, a collaborative nursing team is established to implement comprehensive and holistic nursing interventions for patients, aiming to improve the efficacy and prognosis of patients (11). Diabetes is a chronic metabolic disease with many inducing factors and difficult treatment (12). In addition, the poor physical condition of elderly patients and long-term treatment work can easily lead to adverse reactions, increase the inner negative emotions, and not conducive to the development of later care and treatment (13). MDT nursing for elderly patients with diabetes can ensure that nurses from multiple departments participate in the clinical nursing work of patients, and then ensure the effectiveness and pertinence of nursing measures, promote the quality of nursing as well as improve the prognosis of patients (14).

Studies have shown that intestinal flora can affect human health and is closely linked to the development of diabetes (15). Recently, the effects of dietary fiber on intestinal flora and human health have attracted much attention (16). Dietary fiber is the “seventh nutrient,” closely related to human health, adding high dietary fiber is not only the basis for the treatment of diabetes, but also an effective means to control diet (17). Dietary fiber is divided into insoluble cellulose and soluble cellulose, which has the functions of water absorption, expansion, and encapsulation, and is the matrix for the growth and fermentation of probiotics (18). Insoluble fiber has a strong effect on facilitating intestinal peristalsis, while soluble fiber has a strong adhesion effect and can delay the absorption of sugar (19). Studies have proved that dietary fiber belongs to one of the crucial factors to maintain the normal sensitivity of human tissue cells to insulin, and insufficient intake can result in high incidence of diabetes (20). Besides, it has been documented that increasing dietary fiber intake can reduce fasting blood glucose along with glycosylated hemoglobin levels in diabetic patients, improve insulin sensitivity, and improve insulin resistance (21). Based on drug treatment, long-term supplementation of sufficient dietary fiber can restore the sensitivity of some diabetic patients to normal levels of insulin, and then correct the disorder of glucose metabolism (20). Dietary fiber can delay and decrease the absorption of carbohydrates in the small intestine, so that the body can fully secrete insulin (22). In addition, dietary fiber can be fermented in the large intestine to produce short-chain fatty acids, improve the balance of intestinal flora, and short-chain fatty acids can increase insulin secretion (23). Numerous randomized controlled trials have shown that high dietary fiber intake can improve the diversity of flora and is conducive to the growth of beneficial bacteria such as bifidobacterium (24). Another study has found that dietary fiber can improve the blood sugar level of diabetes patients through the mediating effect of intestinal flora (25). However, the impacts of MDT nursing model combined with high-fiber dietary intervention on blood glucose level and nutritional status in elderly diabetes patients remains unclear.

In this study, we aimed to explore the impacts of MDT nursing model combined with high-fiber dietary intervention on blood glucose level and nutritional status in elderly diabetes patients.

## 2 Methods

### 2.1 Study design

This study was a prospective clinical trial, and the clinical trial registration number was ChiCTR2400088879. One hundred and sixty-eight elderly type 2 diabetes patients admitted from April 2020 to April 2023 were allocated into intervention group and control group using the random number table method, with 84 cases in each group. Inclusion criteria: (1) The patient met the diagnostic criteria for type 2 diabetes as defined by the World Health Organization (WHO) or the American Diabetes Association (ADA), which include typical symptoms of diabetes (such as excessive thirst, excessive hunger, excessive urination, weight loss, etc.), and a random venous plasma glucose level of ≥ 11.1 mmol/L, or a fasting venous plasma glucose level of ≥ 7.0 mmol/L, or a 2-h venous plasma glucose level during the oral glucose tolerance test (OGTT) of ≥ 11.1 mmol/L, or a glycated hemoglobin (HbA1c) level of ≥ 6.5%. Additionally, the patient must have a clear diagnosis record from at least one secondary-level or higher hospital; (2) Age 60–80 years old; multi-disciplinary (3) The duration of diabetes in the included patients should be at least 1 year to ensure that the patients’ conditions were relatively stable and they had a certain level of awareness and self-management ability regarding diabetes. The duration was calculated from the time of the first diagnosis of type 2 diabetes by the patient, up to the time point when they were included in this study; (4) The patients included in this study include those who had not received insulin treatment and were solely treated through diet control, exercise, and oral hypoglycemic drugs; (5) The fasting blood glucose level of the included patients was within the range of 7.0–13.9 mmol/L; (6) The patient’s glycated hemoglobin (HbA1c) level was within the range of 7.0%–10.0%. Exclusion criteria: (1) Patients with severe liver diseases such as liver cirrhosis (judged based on liver morphology changes, abnormal liver function, etc., such as Child-Pugh grade C and above), chronic severe hepatitis (with obvious jaundice, coagulation dysfunction symptoms, total bilirubin ≥ 171 μmol/L, prothrombin activity ≤ 40%), and liver cancer (diagnosed by pathological examination); (2) Patients with stage 4 or higher chronic kidney disease (estimated glomerular filtration rate eGFR < 30 mL/min/1.73 m^2^, calculated according to the formula of the Chronic Kidney Disease Epidemiology Collaboration) or end-stage kidney disease (requiring renal replacement therapy, such as hemodialysis, peritoneal dialysis); (3) Patients with acute myocardial infarction (occurring within 6 months before inclusion in the study), unstable angina pectoris (occurring within 1 month before inclusion in the study), severe arrhythmias (such as ventricular tachycardia, ventricular fibrillation, high-degree atrioventricular block, requiring drug treatment or pacemaker implantation) or heart failure (NYHA cardiac function class III-IV) with serious cardiac diseases; (4) Patients with severe hematological diseases such as leukemia (diagnosed through bone marrow puncture), aplastic anemia (with pancytopenia, low or severely low bone marrow hyperplasia), and myelodysplastic syndrome (with hematopoietic disorders and specific changes in bone marrow morphology); (5) Patients with severe mental disorders such as schizophrenia, bipolar disorder, major depression, and anxiety disorders that significantly affect their cognitive and behavioral abilities will be excluded. The diagnostic criteria are based on the relevant standards in the “International Classification of Diseases, Tenth Edition” (ICD-10) or the “Diagnostic and Statistical Manual of Mental Disorders, Fifth Edition” (DSM-5).

**Figure 1:**
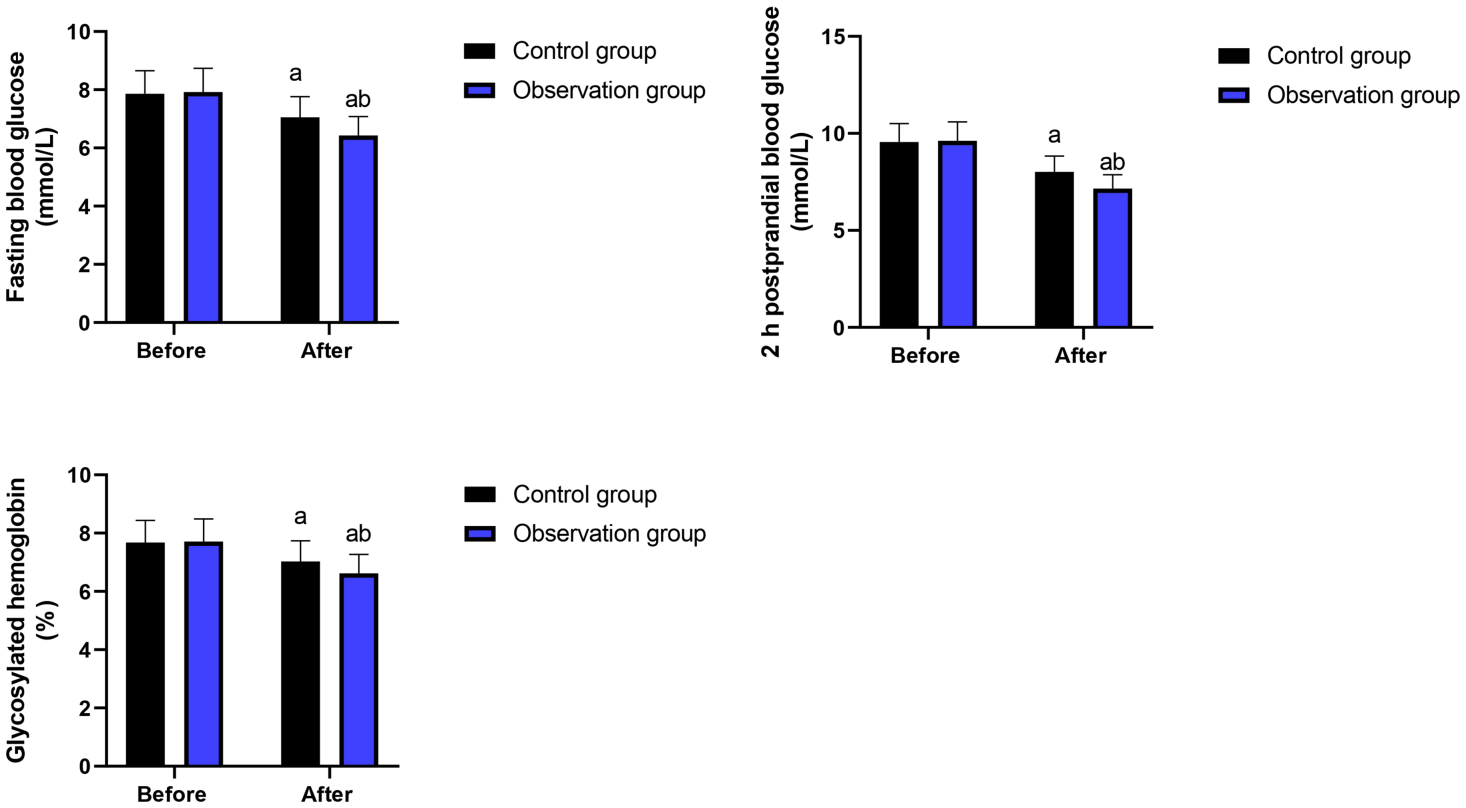
Changes of blood glucose indexes in 2 groups. ^a^*p* < 0.05, compared with before intervention, ^b^*p* < 0.05, compared with CG.

**Figure 2:**
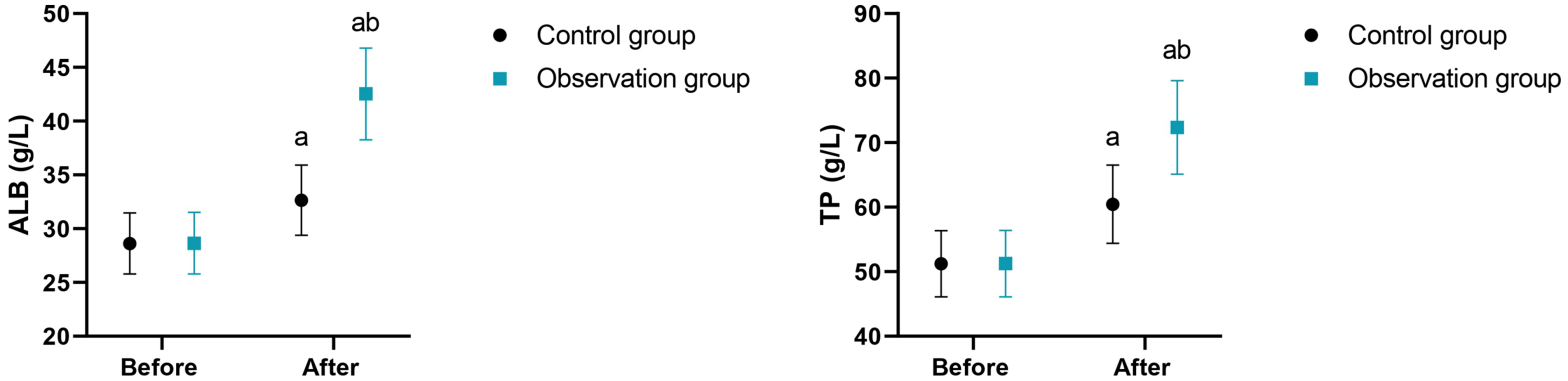
Nutritional status of 2 groups. ^a^*p* < 0.05, compared with before intervention, ^b^*p* < 0.05, compared with CG.

**Figure 3:**
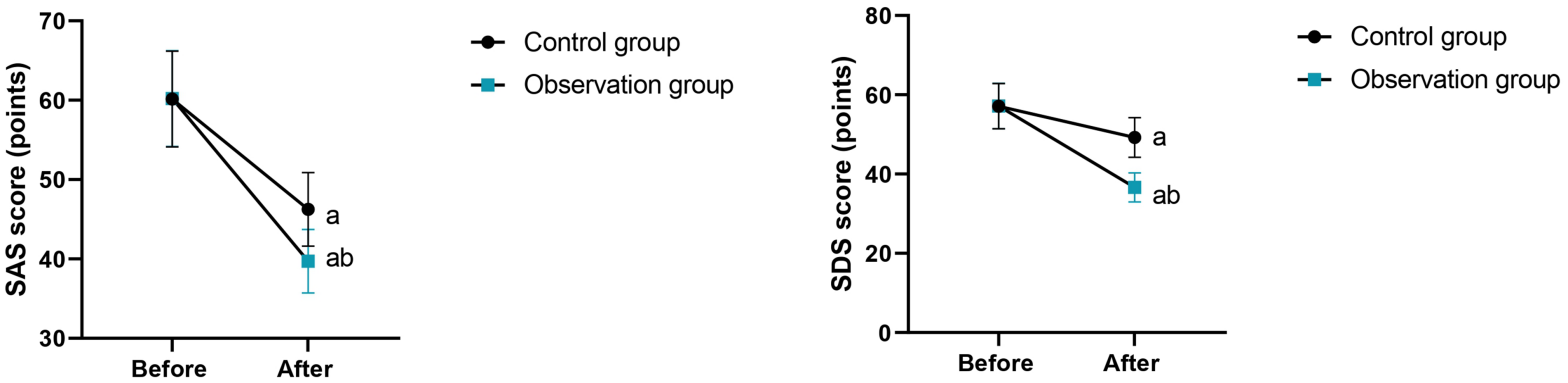
Negative emotions in 2 groups. ^a^*p* < 0.05, compared with before intervention, ^b^*p* < 0.05, compared with CG.

**Figure 4:**
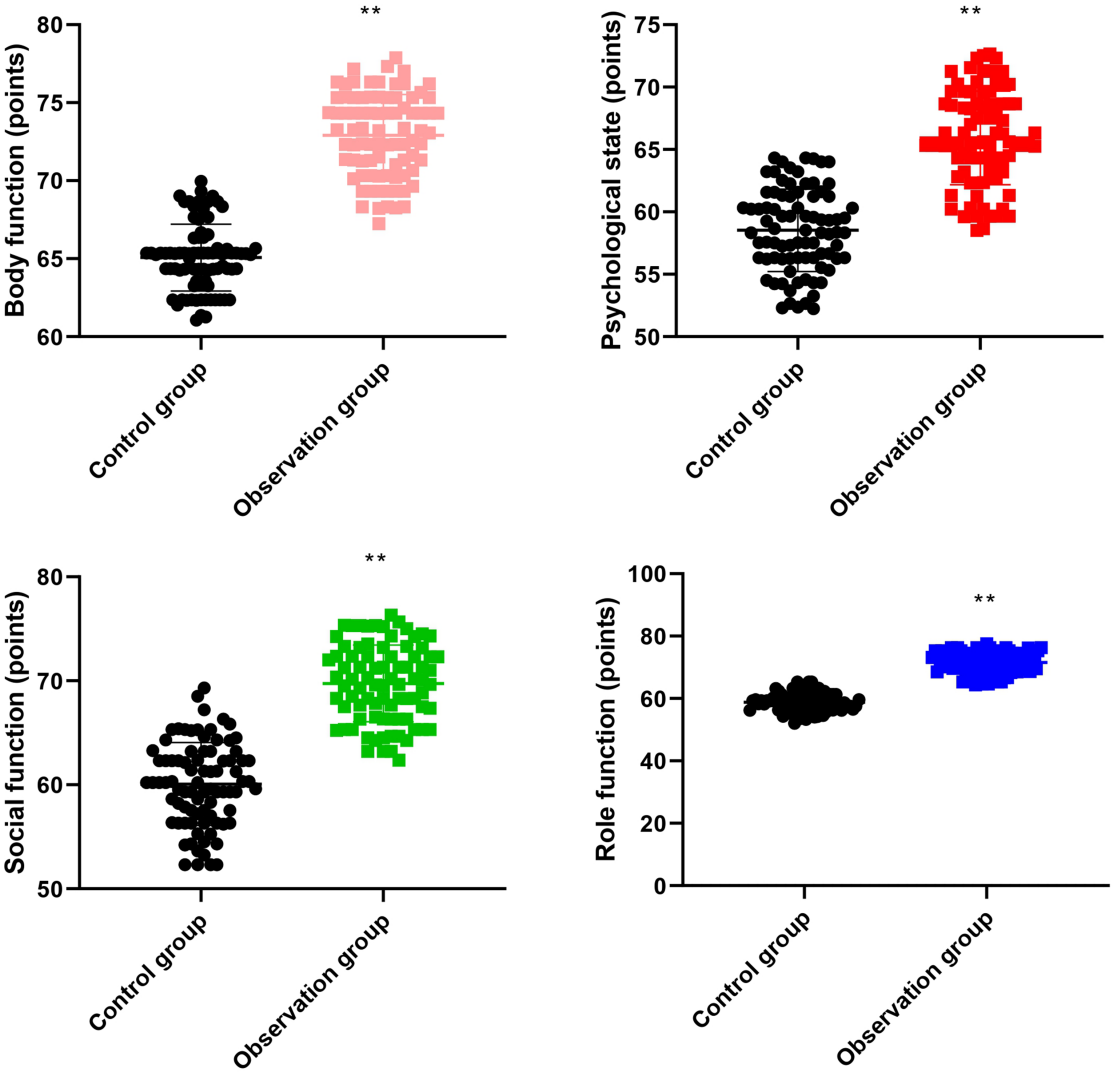
Quality of life in 2 groups. **p < 0.01.

**Figure 5:**
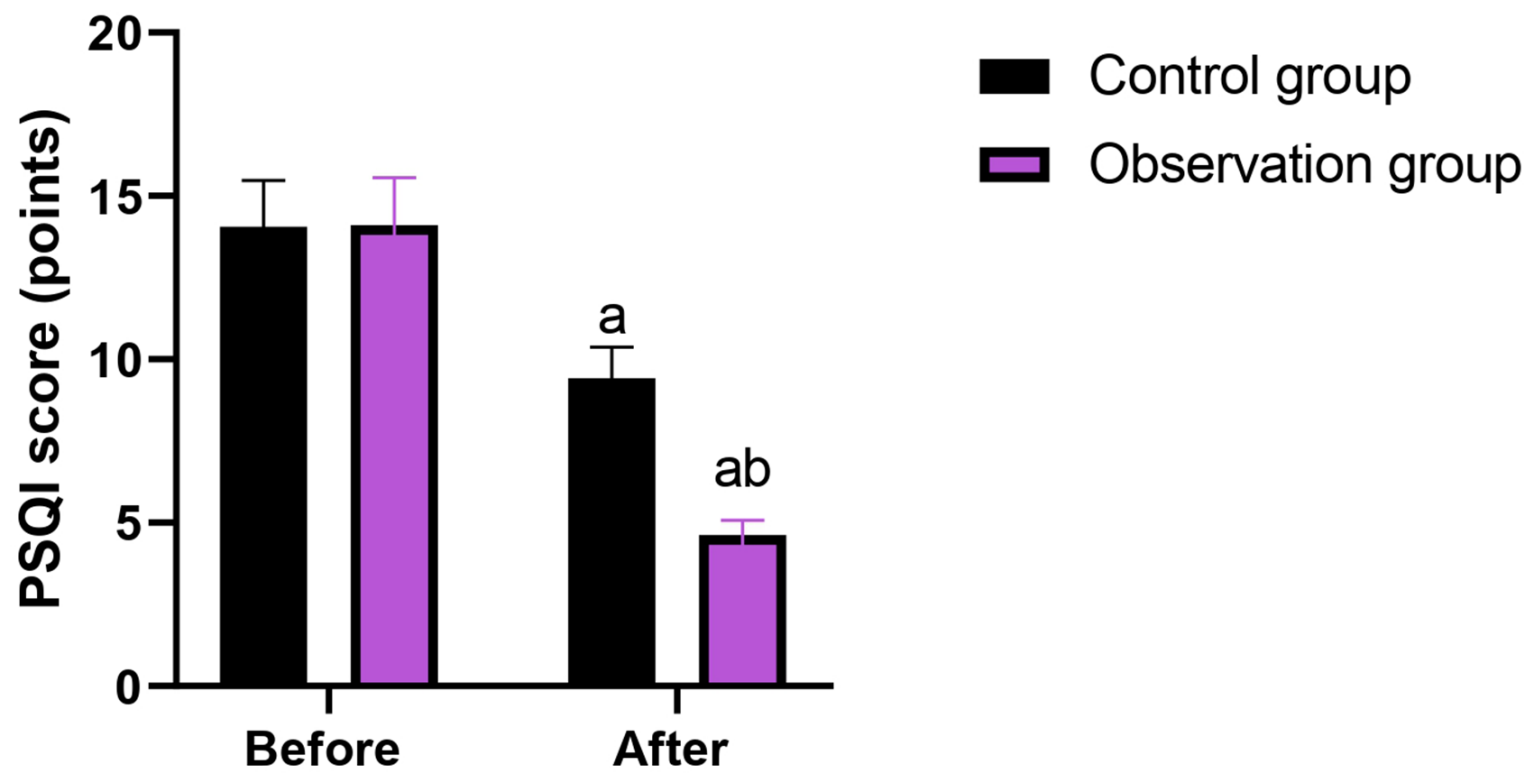
Sleep quality in 2 groups. ^a^*p* < 0.05, compared with before intervention, ^b^*p* < 0.05, compared with CG.

All participants were required to undergo laboratory tests, including liver function tests (such as alanine aminotransferase, aspartate aminotransferase, total bilirubin, albumin) and kidney function tests (such as serum creatinine, urea nitrogen, estimated glomerular filtration rate). For patients with abnormal liver function or suspected liver diseases, further liver ultrasound examination or liver CT/MRI examination was conducted; for patients with abnormal kidney function or suspected kidney diseases, kidney ultrasound examination was performed, and in some cases, a kidney biopsy was carried out for a clear diagnosis. All participants were required to undergo routine electrocardiogram (ECG) tests. For patients with cardiac symptoms or abnormal ECG results, further echocardiography was conducted to assess the structure and function of the heart. If necessary, dynamic electrocardiogram monitoring, coronary angiography, and other examinations were carried out to make a clear diagnosis. All participants were required to undergo a blood routine test. For those with abnormal blood routine results or suspected of having blood system diseases, further tests such as bone marrow puncture and bone marrow biopsy were conducted to make a clear diagnosis. Through detailed inquiries about the patient’s mental health history, mental status examination, and necessary psychological assessment scales, the participants were screened for mental disorders. For patients who suspected they had mental illnesses, they would go to a mental health hospital for further diagnosis and treatment (Figures 1–5).

All patients signed the informed consent. This study was proved by the Ethics Committee of Beijing Friendship Hospital, Capital Medical University, and the approval number was 2020-P2-271.

### 2.2 Randomization

A group randomization design was adopted for random grouping. The random allocation sequence was generated by a computer. The allocation confidentiality measures were achieved through sequential numbering, sealing, and opaque envelopes. After being deemed to meet the inclusion criteria, patients were randomly assigned to the control group or the intervention group in a 1:1 ratio.

### 2.3 Routine nursing and MDT nursing methods

Patients in the control group accepted routine nursing care, and the nursing staff took corresponding nursing measures according to the nursing routine of the department, explained the basic knowledge of diabetes for patients in detail, analyzed and evaluated the cultural literacy of patients, formulated individualized publicity brochures for patients, regularly monitored the blood sugar level, and told patients to take medication on time and according to the doctor’s advice. Besides, the nursing staff adjusted the dosage of oral hypoglycemic drugs (metformin hydrochloride tablet, Chongqing Conquer Pharmaceutical Co., Ltd) according to the actual condition of patients and blood glucose monitoring results.

On the basis of the control group, patients in the intervention group adopted MDT nursing.

(1) Setting up a specialist nursing team: Dietitians, specialist nursing members, vascular and psychiatric medical staff, geriatric nursing specialist nurses were invited to set up a multidisciplinary nursing team, and the team members worked together to formulate a health education schedule, clarify the daily health education time, content and responsible person, and ensure the active implementation of relevant nursing measures.
(2) Health publicity and education: The WeChat public account of the department was set up, patients were guided to scan the code for attention, and diabetes rehabilitation knowledge was regularly pushed. Knowledge was disseminated through the WeChat official account, with an average of 3–4 pieces of content being pushed out each week. Each piece of content took about 10–15 min to read. During the entire intervention period (8 weeks), the total study time of patients through the official account was approximately 2 h. In addition, team members made a graphic publicity and education manual to guide patients to learn the content of the publicity manual by themselves, and responsible nursing staff explained it one-on-one to promote patients to fully grasp the knowledge of disease rehabilitation. On average, each patient spent 1.5 h completing the manual study and comprehension. Based on factors such as the patient’s educational level, age, and cognitive ability, the educational content and methods were adjusted. For patients with lower educational levels and older age, simple and understandable language, as well as illustrated promotional materials, were used for education; for patients with higher educational levels and stronger acceptance abilities, more in-depth and professional knowledge materials were provided, and relevant academic literature was recommended for their study. At the same time, according to the patient’s interests and hobbies, the education form that was more easily accepted by them was selected. For example, for patients who liked watching videos, more video materials were pushed to them; for those who preferred reading text, detailed textual explanations were provided.
(3) Diabetes specialist nursing: By means of text, language, video, health lectures and other forms, diabetes specialists explained the basic knowledge of diabetes to patients and their families in detail, so that patients and their families could fully understand the formation causes, treatment measures, and prognosis of elderly diabetes. A total of 2 health lectures were held, each lasting approximately 1.5 h, with the total duration being 3 h. Diabetes specialists demonstrated hypoglycemic drugs and related precautions to patients and their families, in order to comprehensively improve patients’ disease awareness. Based on the severity of the condition, and the presence of complications of the patient, a personalized diabetes basic knowledge explanation plan was formulated. For patients with complications such as cardiovascular diseases and kidney diseases, the explanation of related complication prevention knowledge was increased to enhance the patient’s overall understanding of the disease.
(4) Multidisciplinary treatment: Cardiovascular and neurology doctors were invited to conduct a comprehensive assessment based on the specific conditions of each patient, such as their heart function and limb function, and to develop personalized rehabilitation exercise plans for each patient. For patients with weaker heart function, the intensity and frequency of rehabilitation exercises were appropriately reduced; for patients with severe limb dysfunction, more targeted rehabilitation training programs were added, such as swallowing function training and limb joint range of motion training. At the same time, based on the changes in the patient’s physical indicators, the rehabilitation plan was adjusted in a timely manner to ensure the safety and effectiveness of the rehabilitation exercises.
(5) Psychological intervention: Psychiatrists conducted comprehensive psychological assessments of each patient using professional tools to understand the patient’s levels of anxiety, depression and other emotions, as well as the root causes of their psychological problems. Based on the assessment results, they discussed with the responsible nursing staff and develop personalized psychological intervention plans for the patients. For patients with mild psychological problems, they used methods such as psychological counseling and health education to help the patients alleviate their emotions; for patients with moderate or more severe psychological problems, they combined psychological treatment techniques, such as cognitive behavioral therapy and relaxation training, for systematic intervention. At the same time, they adjusted the psychological intervention plans in a timely manner according to the changes in the patients’ psychological states to ensure their mental health.
(6) Support methods for patients in terms of follow-up, supervision and question-and-answer sessions: The medical staff communicated with the patients via WeChat on a regular basis to understand the changes in their condition, the progress of their recovery, and their learning of educational materials. They conducted at least two online exchanges with the patients every week to keep track of their current situation in a timely manner. Combining face-to-face consultations and visitation sessions, the medical staff conducted comprehensive follow-ups for patients. During the consultations and visits, the medical staff inquired in detail about the patient’s physical condition, diet, exercise, medication, assessed the patient’s recovery progress, and provided targeted suggestions and guidance to the patients. The medical staff used the data statistics function on the WeChat official account to understand the reading situation of patients regarding the pushed knowledge, including the number of reads and the reading duration. For patients with low reading volumes, the medical staff sent private messages via WeChat to remind them to study and understand the reasons for their non-reading, and provide corresponding assistance. During face-to-face consultations and visits, medical staff checked the patients’ learning progress of the graphic and textual educational brochures. They assessed the patients’ mastery of the knowledge through questioning and having the patients repeat the information. For patients who were not serious about learning, the medical staff provided patient guidance and education, emphasizing the importance of learning. During the patient’s hospitalization, the nursing staff closely monitored whether the patient followed the dietary, exercise and medication recommendations, and promptly corrected any improper behaviors of the patient. For discharged patients, regular inquiries were made through phone calls or WeChat to understand their daily behavior, and reminders were given to them to take their medication on time, have a balanced diet and engage in appropriate exercise. During the face-to-face consultations and visits every 2 weeks, a comprehensive assessment was conducted on the patient’s physical indicators, rehabilitation exercise status, and psychological condition, to determine whether the patient was following the recommendations and the effectiveness of the implemented suggestions. Based on the assessment results, the nursing plan was adjusted promptly to ensure that the patient receives scientific and reasonable care. The questions raised by patients in the WeChat communication group were answered by medical staff immediately. For some common questions, they were compiled into documents and published on the official account to facilitate more patients’ learning. At the same time, a dedicated online Q&A time was set up, with 2 sessions per week, each lasting 1–2 h, during which professional medical staff collectively answered patients’ questions. During face-to-face consultations and visits, patients asked questions at any time to the medical staff. The medical staff provided detailed and patient answers to ensure that the patients understand and master the relevant knowledge. For complex issues, a multidisciplinary team was organized to discuss together and give the patients accurate responses.

Both groups received an 8-week intervention.

### 2.4 Diet intervention methods

Control group: Nutritional assessment was conducted by a dietitian to understand the nutritional status of patients, refer to the target range of blood sugar control, and formulate a diet plan. It is necessary to choose food that is conducive to stable control of blood sugar, respect the individual taste of patients, on this basis, scientific nutrition collocation can achieve personalized healthy diet, meet the nutritional needs of patients, and avoid abnormal rise in blood sugar.

On the basis of the control group, patients in the intervention group were given high-fiber dietary intervention, including corn fiber 38.9%, wheat fiber 19.4%, oat fiber 38.9%, and soy fiber 5.5%, 1 pack/time, twice a day, half an hour before breakfast and dinner, for 8 weeks. Medication method: 150 mL boiled water was added and stir well before taking, and more than 100 mL liquid was added after each taking.

Both groups received an 8-week intervention.

### 2.5 Observation indicators

(1) The changes of fasting blood glucose, 2 h postprandial blood glucose as well as glycosylated hemoglobin were compared between two groups before and 8 weeks after intervention.
(2) The incidence of complications including diabetic foot, pulmonary infection, diabetic retinopathy, and hypoglycemia was recorded in two groups during the 8-week intervention.
(3) The Generic Quality of Life Inventory 74 (GQOL-74) was implemented to evaluate the quality of life of patients 8 weeks after intervention, which contained four aspects: body function, psychological state, social function and role function. The score was proportional to the quality of life (26).
(4) The nutritional status of two groups was compared before and 8 weeks after intervention, including albumin (ALB) and total protein (TP).
(5) The self-rating Anxiety Scale (SAS) together with Self-rating Depression Scale (SDS) were adopted to evaluate the mood of the patients (27) before and 8 weeks after intervention. The full score of the scales was 100 points, and the higher the score, the more anxious and depressed the patients were.
(6) Pittsburgh Sleep Quality Index Scale (PSQI) was implemented to assess the sleep quality of patients (28) before and 8 weeks after intervention. The score scale consisted of 18 items and seven factors. The total score was 0–21 points.
(7) The patient satisfaction of two groups was compared. Assessment was carried out after nursing. Patients rated the overall nursing situation, which was divided into unsatisfactory, satisfactory and very satisfactory. Patient satisfaction = (very satisfied + satisfied) cases/total cases ×100%.

### 2.6 Statistical analysis

SPSS 24.0 statistical software was adopted for data analysis. Measurement data were expressed as (*x* ± *s*), and *t*-test was adopted for comparison. Count data were expressed as (*n*, %), and *χ*^2^ test was used for comparison. *p* < 0.05 meant statistical significance.

## 3 Results

### 3.1 General data of patients between the two groups

In the control group, there were 42 males and 42 females, ranging in age from 63 to 88 years, with an average age of (67.98 ± 6.09) years. The disease course was 2–34 years, with an average age of (13.48 ± 2.12) years. The average length of hospital stay was 10.23 ± 1.05, ranging from 7 to 13 days. The intervention group included 44 males and 40 females, ranging in age from 64 to 89 years, with an average age of (68.27 ± 7.28) years, and the disease course from 2 to 35 years, with an average age of (13.52 ± 1.98) years. The average length of hospital stay was 10.15 ± 1.02, ranging from 7 to 14 days. No difference was discovered in general data between two groups (*p* > 0.05, Table 1).

**Table 1:** General data of patients between the two groups.

Items	Control group (*n* = 84)	Intervention group (*n* = 84)	*χ*^2^/*t*	*p*
Gender			0.095	0.757
Male	42 (50.00)	44 (52.38)		
Female	42 (50.00)	40 (47.62)		
Age (years)	67.98 ± 6.09	68.27 ± 7.28	0.280	0.779
Course of disease (years)	13.48 ± 2.12	13.52 ± 1.98	0.126	0.899
Average length of hospital stay (days)	10.23 ± 1.05	10.15 ± 1.02	0.500	0.617

### 3.2 Changes of blood glucose indexes between the two groups

Before intervention, there were no differences in fasting blood glucose [(7.86 ± 0.79) mmol/L vs. (7.92 ± 0.82) mmol/L], 2 h postprandial blood glucose [(9.56 ± 0.95) mmol/L vs. (9.62 ± 0.97) mmol/L] and glycosylated hemoglobin [(7.68 ± 0.76) % vs. (7.71 ± 0.78) %] between the two groups (*p* > 0.05). After 8 weeks of intervention, the fasting blood glucose, 2 h postprandial blood glucose and glycosylated hemoglobin in the intervention group were (6.43 ± 0.65) mmol/L, (7.15 ± 0.72) mmol/L and (6.62 ± 0.65) %, respectively, and those in the control group were (7.05 ± 0.71) mmol/L, (8.02 ± 0.81) mmol/L and (7.02 ± 0.73) %, respectively. Compared with the control group, the intervention group had lower fasting blood glucose, 2 h postprandial blood glucose and glycosylated hemoglobin after 8 weeks of intervention (*p* < 0.05, Table 2).

**Table 2:** Changes of blood glucose indexes between the two groups.

Items	Time	Control group (*n* = 84)	Intervention group (*n* = 84)	*t*	*p*
Fasting blood glucose (mmol/L)	Before intervention	7.86 ± 0.79	7.92 ± 0.82	0.783	0.629
	8 weeks after intervention	7.05 ± 0.71	6.43 ± 0.65	5.903	<0.001
*t*		6.989	13.050		
*p*		<0.001	<0.001		
2 h postprandial blood glucose (mmol/L)	Before intervention	9.56 ± 0.95	9.62 ± 0.97	0.405	0.686
	8 weeks after intervention	8.02 ± 0.81	7.15 ± 0.72	7.357	<0.001
*t*		11.305	18.739		
*p*		<0.001	<0.001		
Glycosylated hemoglobin (%)	Before intervention	7.68 ± 0.76	7.71 ± 0.78	0.252	0.801
	8 weeks after intervention	7.02 ± 0.73	6.62 ± 0.65	3.750	0.000
*t*		5.740	9.839		
*p*		<0.001	<0.001		

### 3.3 Incidence of complications between the two groups

The total incidence rate of complications in the control group was 14.28% (12/84), and that in the intervention group was 4.76% (4/84). In contrast to the control group, the incidence of complications in the intervention group was lower (*p* < 0.05, Table 3).

**Table 3:** Incidence of complications between the two groups.

Groups	*N*	Diabetic foot	Pulmonary infection	Diabetic retinopathy	Hypoglycemia	Total incidence rate
Control group	84	3 (3.57)	2 (2.38)	3 (3.57)	4 (4.76)	12 (14.28)
Intervention group	84	1 (1.19)	1 (1.19)	1 (1.19)	1 (1.19)	4 (4.76)
*χ* ^2^						4.421
*p*						0.035

### 3.4 Quality of life between the two groups

After 8 weeks of intervention, the scores of body function, psychological state, social function and role function in the intervention group were (73.58 ± 7.42) points, (67.69 ± 6.82) points, (69.87 ± 7.03) points and (70.68 ± 7.09) points, respectively, while those in the control group were (68.59 ± 7.02) points, (58.69 ± 6.05) points, (62.58 ± 6.32) points and (61.90 ± 6.32) points, respectively. Compared with the control group, the intervention group had higher scores of body function, psychological state, social function and role function (*p* < 0.01, Table 4).

**Table 4:** Quality of life between the two groups.

Items	Control group (*n* = 84)	Intervention group (*n* = 84)	*t*	*p*
Body function	68.59 ± 7.02	73.58 ± 7.42	4.477	<0.001
Psychological state	58.69 ± 6.05	67.69 ± 6.82	9.047	<0.001
Social function	62.58 ± 6.32	69.87 ± 7.03	7.067	<0.001
Role function	61.90 ± 6.32	70.68 ± 7.09	8.472	<0.001

### 3.5 Nutritional status between the two groups

Before intervention, there were no differences in ALB [(28.62 ± 2.84) g/L vs. (28.65 ± 2.87) g/L] and TP [(51.23 ± 5.12) g/L vs. (51.25 ± 5.16) g/L] between the two groups (*p* > 0.05). After 8 weeks of intervention, the levels of ALB and TP in the intervention group were (42.53 ± 4.26) g/L and (72.36 ± 7.25) g/L, respectively, and those in the control group were (32.65 ± 3.26) g/L and (60.45 ± 6.05) g/L, respectively. Compared with the control group, the intervention group had higher ALB and TP levels (*p* < 0.05, Table 5).

**Table 5:** Nutritional status between the two groups.

Items	Time	Control group (*n* = 84)	Intervention group (*n* = 84)	*t*	*p*
ALB (g/L)	Before intervention	28.62 ± 2.84	28.65 ± 2.87	0.068	0.945
	8 weeks after intervention	32.65 ± 3.26	42.53 ± 4.26	16.880	<0.001
*t*		8.542	24.765		
*p*		<0.001	<0.001		
TP (g/L)	Before intervention	51.23 ± 5.12	51.25 ± 5.16	0.025	0.979
	8 weeks after intervention	60.45 ± 6.05	72.36 ± 7.25	11.559	<0.001
*t*		10.661	21.741		
*p*		<0.001	<0.001		

### 3.6 Negative emotions and sleep quality between the two groups

Before intervention, there were no differences in SAS score [(60.15 ± 6.02) points vs. (60.21 ± 6.04) points], SDS score [(57.12 ± 5.71) points vs. (57.16 ± 5.74) points] and PSQI score [(14.05 ± 1.42) points vs. (14.10 ± 1.46) points] between the two groups (*p* > 0.05). After 8 weeks of intervention, the SAS score, SDS score and PSQI score in the intervention group were (39.71 ± 4.01) points, (36.65 ± 3.68) points and (4.62 ± 0.46) points, respectively, and those in the control group were (46.25 ± 4.62) points, (49.23 ± 5.01) points and (9.42 ± 0.95) points, respectively. Compared with the control group, the intervention group had lower SAS score, SDS score and PSQI score (*p* < 0.05, Table 6).

**Table 6:** Negative emotions and sleep quality between the two groups.

Items	Time	Control group (*n* = 84)	Intervention group (*n* = 84)	*t*	*p*
SAS score (points)	Before intervention	60.15 ± 6.02	60.21 ± 6.04	0.064	0.948
	8 weeks after intervention	46.25 ± 4.62	39.71 ± 4.01	9.798	<0.001
*t*		16.788	25.915		
*p*		<0.001	<0.001		
SDS score (points)	Before intervention	57.12 ± 5.71	57.16 ± 5.74	0.045	0.963
	8 weeks after intervention	49.23 ± 5.01	36.65 ± 3.68	18.547	<0.001
*t*		9.519	27.569		
*p*		<0.001	<0.001		
PSQI score (points)	Before intervention	14.05 ± 1.42	14.10 ± 1.46	0.225	0.822
	8 weeks after intervention	9.42 ± 0.95	4.62 ± 0.46	41.679	<0.001
*t*		24.837	56.760		
*p*		<0.001	<0.001		

### 3.7 Total satisfaction rate of patients between the two groups

The total satisfaction rate of the control group was 80.95% (68/84), and that of the intervention group was 94.05% (79/84). In contrast to the control group, the total satisfaction rate of patients in the intervention group was better (*p* < 0.05, Table 7).

**Table 7:** Patient satisfaction in two groups.

Groups	*N*	Very satisfied	Satisfied	Dissatisfied	Total satisfaction rate
Control group	84	40 (47.62)	28 (33.33)	16 (19.05)	68 (80.95)
Intervention group	84	45 (53.57)	34 (40.48)	5 (5.95)	79 (94.05)
*χ* ^2^					6.585
*p*					0.010

## 4 Discussion

Older patients with diabetes often have multiple chronic diseases, with their physical functions deteriorating and metabolic capabilities weakening. They not only require comprehensive medical care, but also need an intervention method that can effectively improve their metabolic conditions and enhance their quality of life (29). The MDT nursing model can provide various forms of support, but it lacks direct and effective intervention measures for the increasingly prominent nutritional issues and the large fluctuations in blood sugar levels of elderly patients with diabetes.

The intervention of a high-fiber diet can directly affect the glucose metabolism of diabetic patients. Dietary fiber can delay the absorption of carbohydrates, reduce the rate of post-meal blood sugar increase, and increase satiety and reduce food intake, which is helpful for controlling weight and blood sugar levels (30). Combining the MDT nursing model with the high-fiber diet intervention can exert the synergistic effect of the two. The nutritionist in the MDT team can, based on the specific conditions of the patients, formulate personalized high-fiber diet plans to ensure that patients consume sufficient dietary fiber while maintaining a balanced nutrition. Other professional personnel from other disciplines, such as doctors, nurses, and rehabilitation therapists, can provide support and guidance within their respective professional fields to help patients better implement the diet plan and improve the intervention effect.

Therefore, in this study, we did not solely adopt the MDT nursing model, but combined it with a high-fiber diet intervention measure to explore the impact of the combination of the MDT nursing model and the high-fiber diet intervention on the blood sugar levels and nutritional status of elderly diabetic patients.

The results indicated that after 8 weeks of intervention, in contrast to the control group, the fasting blood glucose, 2 h postprandial blood glucose and glycosylated hemoglobin in the intervention group were lower, and the incidence of complications in the intervention group was lower (Tables 2, 3) suggesting that MDT model combined with high-fiber dietary intervention could effectively control blood glucose level as well as reduce the incidence of complications in elderly patients with diabetes. Consistently, it has been reported that MDT integrated management combined with all-media health education intervention can effectively ameliorate blood glucose, blood pressure, and blood lipids of patients with coronary heart disease and diabetes mellitus, promote their healthy life, improve their self-efficacy, and improve their negative emotions and quality of life (31). However, this study also has some differences from earlier studies. In terms of the intervention methods, this study places greater emphasis on the collaboration of multidisciplinary teams and precise intervention with high-fiber diets. The various professionals in the MDT team, such as endocrinologists, nutritionists, nurses, and rehabilitation therapists, work together to develop personalized intervention plans based on the specific conditions of the patients, ensuring the targeted and effective nature of the intervention measures.

Moreover, our study indicated that after 8 weeks of intervention, the scores of body function, psychological state, social function as well as role function in the intervention group were higher as comparing with the control group, the SAS and SDS scores in the intervention group were lower as comparing with the control group, the PSQI score in the intervention group was lower as comparing with the control group, and the patient satisfaction in the intervention group was higher as comparing with the control group (Tables 4, 6, 7), suggesting that MDT model combined with high-fiber dietary intervention could effectively promote the quality of life, relieve negative emotions, promote the sleep quality along with promote patient satisfaction in elderly patients with diabetes. In line with our findings, Liu et al. (32) suggested that the application of the multidisciplinary collaborative team combined with tranquilisation therapy in patients with terminal cancer can significantly reduce the anxiety and depression of patients, enable patients to obtain comprehensive social support, and effectively improve the quality of life of patients. Similarly, Han et al. performed a randomized controlled trial and discovered that the online multidisciplinary weight loss management program improve blood glucose, promote self-management ability and improve the quality of life in obese or overweight patients living with type 2 diabetes mellitus (33). Compared with previous studies, this research differs in the details of the intervention content. The high-fiber diet intervention in this study not only focuses on the intake of dietary fiber, but also, based on the patients’ taste preferences, dietary habits, and nutritional needs, a personalized diet plan was formulated, which enhanced the patients’ compliance with the dietary intervention. While earlier studies might have described the specific content of the dietary intervention relatively less and lacked personalized design considerations. Moreover, the MDT team in this study paid more attention to communication and interaction with the patients during the intervention process, promptly understanding the patients’ needs and feedback, adjusting the intervention plan, and enhancing the patients’ confidence and satisfaction in the treatment. The earlier studies might have paid insufficient attention to this aspect, resulting in a less significant improvement in patient satisfaction compared to this study.

Additionally, our study indicated that after 8 weeks of intervention, the ALB and TP levels in the intervention group were higher as comparing with the control group (Table 5), suggesting that MDT model combined with high-fiber dietary intervention could effectively promote the nutritional status in elderly patients with diabetes. Consistently, He et al. (34) proposed that multidisciplinary team cooperation can significantly improve the levels of nutritional indexes including hemoglobin, serum albumin, and transferrin of stroke patients with hemiplegia. This study differs from the research conducted by He et al. (34) in terms of the research subjects and intervention methods. The research subjects of this study are elderly diabetic patients. These patients have declined physical functions, and their nutritional needs and metabolic characteristics are different from those of general diabetic patients. This study has formulated a more suitable intervention plan for elderly patients based on their characteristics. For example, the texture and quantity of the diet are adjusted to adapt to the chewing and digestion abilities of elderly patients. While the research conducted by He et al. (34) may not have fully considered the particularities of elderly patients, the targetedness of the intervention plan is relatively weak. Moreover, the collaboration of the MDT team in this study played a significant role in the nutritional intervention. Dietitians worked closely with professionals from other disciplines to jointly monitor the patients’ nutritional status and overall health, ensuring the comprehensiveness and effectiveness of the intervention measures. The research conducted by He et al. (34) may lack this multi-disciplinary collaboration model, and the intervention effect may be limited.

Our research has some limitations. Firstly, our sample size is relatively small, which may lead to deviations between the data results and the actual values. Secondly, our research was a single-center study, and the sample was not representative, which may not accurately reflect the characteristics of a broader population. Thirdly, our research only conducted 8-week observations. The effects of MDT model combined with high-fiber dietary intervention on the long-term blood glucose level and nutritional status of elderly diabetes patients are currently unclear. Moreover, this study did not monitor and analyze the average insulin dosage of the two groups of patients before and after the intervention. Future research will improve the monitoring and analysis of this indicator to explore the comprehensive effect of the intervention measures in greater depth. Finally, this study did not conduct a systematic monitoring and analysis of hypoglycemic episodes and the adverse reactions caused by a high-fiber diet. These two aspects are of great significance in the treatment and management of elderly diabetic patients. Their omission from the consideration may lead to certain limitations in the comprehensive reflection of the overall impact of the intervention measures. Future studies will improve the monitoring of relevant indicators to more comprehensively evaluate the effectiveness of the intervention measures. Therefore, more multi-center, large-scale, and long-term studies should be conducted in the future to further verify our findings.

In conclusion, our study clarifies that MDT model combined with high-fiber dietary intervention can control blood glucose level, promote nutritional status, decrease the incidence of complications as well as promote the quality of life in elderly diabetes patients.

## Data Availability

The datasets presented in this study can be found in online repositories. The names of the repository/repositories and accession number(s) can be found in the article/supplementary material.
